# Surgical counting interruptions in operating rooms

**DOI:** 10.1186/s12912-024-01912-1

**Published:** 2024-04-10

**Authors:** Zhi Lujun, Gao Yuan, Wang Wei

**Affiliations:** 1 Anesthesia&Operation Center, West China Hospital, Sichuan University, Chengdu, China; 2 West China Hospital, Sichuan University, No. 37 Guoxue Xiang, Wuhou District, 610041 Chengdu, Sichuan China

**Keywords:** Surgical count, Interruption, Operating room nursing, Hospital nursing staff

## Abstract

**Background:**

Operating rooms are complex working environments with high workloads and high levels of cognitive demand. The first surgical count which occurs during the chaotic preoperative stage and is considered a critical phase, is a routine task in ORs. Interruptions often occur during the first surgical count; however, little is known about the first surgical counting interruptions. This study aimed to observe and analyse the sources, outcomes, frequency of the first surgical counting interruptions and responses to interruptions.

**Methods:**

A retrospective observational study was carried out to examine the occurrence of the first surgical counting interruptions between 1st August 2023 and 30th September 2023. The data were collected using the “Surgical Counting Interruption Event Form”, which was developed by the researchers specifically for this study.

**Results:**

A total of 66 circulating nurses (CNs) and scrub nurses (SNs) were observed across 1015 surgeries, with 4927.8 min of surgical count. The mean duration of the first surgical count was 4.85 min, with a range of 1.03 min to 9.51 min. In addition, 697 interruptions were identified, with full-term interruptions occurring an average of 8.7 times per hour. The most frequent source of interruption during the first surgical counts was instruments (*N* = 144, 20.7%). The first surgical counting interruptions mostly affected the CN (336 times; 48.2%), followed by the ORNs (including CNs and SNs) (243 times; 34.9%) and the SN (118 times; 16.9%). Most of the outcomes of interruptions were negative, and the majority of the nurses responded immediately to interruptions.

**Conclusions:**

The frequency of the first surgical counting interruption is high. Managers should develop interventions for interruptions based on different surgical specialties and different nursing roles.

**Supplementary Information:**

The online version contains supplementary material available at 10.1186/s12912-024-01912-1.

## Introduction

The operating room is characterized by high workloads, advanced technology, and the involvement of multiple and interdependent medical specialties. Furthermore, operating rooms are error-prone environments. Therefore, the Association of Operating Room Nurses (AORN) recommends that nurses implement no interruption zones in the perioperative environment when performing processes in critical phases that require concentration to avoid distractions and errors [1]. Surgical counting is an important procedure for ensuring the safety of patients and the occupational health of medical staff. Therefore, surgical counting is a critical phase.

According to the guidelines proposed by the Chinese Nursing Association (CNA) and AORN, the standard surgical count procedure requires at least four counts, as follows: first, before the start of the surgery; second, before closing the body cavity; third, after closing the body cavity; fourth, after surgery. The first surgical count is the most critical phase and is the basis for the subsequent three counts. It occurs during the busy and chaotic preoperative stage, and OR nurses are responsible for providing high-quality perioperative care and supporting anaesthetic-surgical interventions by providing necessary supplies and surgical equipment and monitors, as requested by the OR team. Therefore, interruptions often occur.

Interruption refers to a reaction triggered by external stimuli or secondary activities that interrupt focused concentration on a primary task, thus leading to task switching or concurrent multitasking [2, 3]. The definition includes the source, attributes, outcomes and responses. The attributes of interruptions are classified as intrusions, distractions, discrepancies, and breaks [3]. The outcomes of interruptions can be either positive or negative. The responses to interruptions are classified as immediate interruptions, slightly delayed, multitasking and refused interruptions. Currently, most related studies have examined the negative aspects of interruptions [4, 5]. Surgical counting interruptions may result in prolonged operating time, reduced risk detection capabilities, and increased mental workload [6–8]. Therefore, measures should be taken to respond to interruptions, which should be managed according to source, attributes, outcomes and responses.

Through a literature search, we found that current interruption studies have focused mainly on interruptions in the ICU and in the ward [4, 9]. Few studies have focused on interruptions in the OR, and these studies have focused on interruptions during the entire surgical procedure. Fewer studies have focused on surgical count. We therefore observed and analyse the sources, attributes, outcomes of the first surgical counting interruptions and responses to interruptions with the aim of investigating the frequency of the first counting interruptions and frequency of circulating nurses and scrub nurses affected by interruptions.

## Methods

This study was approved by the ethics committee of the study hospital (West China Hospital of Sichuan University (No. 209)). This was a retrospective observational study. The study observed and analysed the sources, attributes, outcomes and frequency of the first surgical counting interruptions and responses to interruptions by reviewing surveillance video recordings of ORs at West China Hospital, Sichuan University, which is a national treatment centre for severe and complicated cases in Southwest China, from 1st August 2023 to 30th September 2023. The study included OR teams from four surgical specialties, namely, cardiovascular surgery, thoracic surgery, neurosurgery, and plastic surgery. Emergency surgery was excluded because of the relative flexibility of staffing. Study participants were selected from a total of 147 nurses by simple random sampling. This data consisted of 1,203 patients, of which 188 were excluded due to unclear or incomplete surveillance videos or data from procedures performed under local anaesthesia. As a result, 1,015 surgeries were included in the data analysis.

The observed preoperative stage for each patient included the time from the start of the first surgical count to its end; moreover, any discrepancy was checked after all counts were complete and surgery was over. The hours of observation per day were 8 h. Before the survey, we reviewed domestic and foreign literature and then designed a surgical counting interruption event form. Then, experts were consulted to test the content validity and develop a revised version of the form. Six experts assessed the relevance of the items in the first draft of the table and suggested revisions. We calculated the item content validity index of the form (i.e., 0.83–1) and the content validity index of the complete observation form (i.e., 0.83). The revised version of the form was used to test surveillance video recordings for 20 surgeries (i.e., 102.21 min), after which the final version of the observation form was developed. Cronbach’s α coefficient of the complete observation form was 0.71. The observation form included general information such as participants’ demographics (gender, age, education, and years of working experience), the first surgical count duration, and attributes, outcomes of the first surgical counting interruptions and responses to interruptions. The sources of interruption were classified as follows:

1. people entering or exiting the OR (e.g., borrowing or returning something, requesting help);
2. surgeons (e.g., informing CNs to prepare the special surgical instruments or other supplies);
3. anaesthetists (e.g., asking CNs to intervene when the intravenous injection is too slow, asking CNs about the order of surgical medication, asking CNs for supplies);
4. instruments (difficulty in checking the integrity of microsurgical instruments, e.g., tips of microsurgical scissors, the small assembly screws which are built-in);
5. disinfection supply centre (e.g., not closing the tip of the clamp neatly, order of instrument string does not match instrument count paper);
6. procedure (interruptions intrinsic to surgical work, e.g., ring forceps and other materials are required for sterilization of surgical sites during the first surgical count).
7. environment (e.g., the ringing of the fixed-line telephone in the OR, noises from equipment alarms, messy operation tables, overly loud music, noise outside the OR);
8. electrophysiological monitoring staff (e.g., requesting to record number of electrodes).
9. nurses themselves (e.g., surgical count was too fast, CNs asked to check again, discrepancies between surgical count and instrument count paper, teaching SNs at the start of the learning curve).

Prior to the formal observation, the researcher selected and trained two observers. The criteria of the observers were as follows: (1) had a registered nurse with > 5 years of working experience in OR, (2) were familiar with the surgical specialties involved in this study, and (3) did not participate in the operation during the observational period. The observers were trained on the requirements for surveillance video data and the concept of interruption, and they correctly interpreted the content of each observation index and key component in the observation. To ensure the objectivity and accuracy of the collected data, two observers simultaneously reviewed surgical surveillance video for 20 surgeries using the “Surgical Counting Interruption Form”. Any disagreements were resolved by discussion to ensure consistency. Interrater reliability was calculated between the two observers, with a kappa coefficient of 0.81.

### Data analysis

Statistical analysis was conducted using SPSS 22.0 software. To ensure data accuracy and integrity, the original data were entered and checked by two researchers. The frequency and constituent ratio were used for statistical description, chi-square tests were used for the comparison of categorical data, and analysis of variance (ANOVA) was used for the comparison of continuous variables. *P* < 0.05 was considered to indicate statistical significance.

## Results

### Participants’ demographics

Among 66 circulating nurses (CNs) and scrub nurses (SNs), 60 (90.9%) were female, and 6 (9.1%) were male, 62 (93.9%) were bachelor’s degree or below, and 4 (6.1%) were graduates, 0–5 years of clinical practice were 9 (13.6%), 5–10 years of clinical practice were 45 (68.2%), and > 10 years of clinical practice were 12 (18.2%) (Table 1).

**Table 1:** Characteristics of study sample

Characteristic		Number	Percentage (%)
Gender			
	Male	6	9.1
	Female	60	90.9
Age, y			
	21–30	30	45.4
	31–40	25	37.9
	> 40	11	16.7
Educational level			
	≥graduate	4	6.1
	≤bachelor’s degree	62	93.9
Years of clinical practice, y			
	0–5	9	13.6
	5–10	45	68.2
	> 10	12	18.2
Overall		66	100

1015 surgical counts were performed within 82 h and 7.8 min. The mean surgical count duration from the start to the end of the first surgical count was 4.85 min, with a range of 1.03 min to 9.51 min. No discrepancies were found after all counts were complete and surgery was complete. The study included OR teams from four surgical specialties, namely, cardiovascular surgery (*N* = 232, 22.86%), thoracic surgery (*N* = 232,2 2.86%), neurosurgery surgery (*N* = 348, 34.28%) and plastic surgery (*N* = 203, 20.0%). A total of 697 interruptions were identified. This means that the full term was interrupted 8.7 times per hour on average. Table 2 presents the total counts and their interruption sources from 1015 surgical counts. Most of the observed interruptions were caused by instruments (*N* = 144,20.7%). The remaining interruptions were attributed to the procedure (*N* = 120, 17.2%) or to the disinfection supply centre (*N* = 117, 16.8%) (Table 2).

**Table 2:** Frequency of different sources of the first surgical counting interruptions

Scource	Description of the interruption	Frequency	Percentage (%)
People entering or exiting the OR	borrowing or returning something, requesting help	93	13.3
Surgeons	informing CNs to prepare the special surgical instruments or other supplies	32	4.6
Anaesthetists	asking CNs to intervene when the intravenous injection is too slow, asking CNs about the order of surgical medication, asking CNs for supplies	35	5.0
Instruments	difficulty in checking the integrity of microsurgical instruments	144	20.7
Disinfection supply center	not closing the tip of the clamp neatly, order of instrument string doesn’t match instrument count paper	117	16.8
Procedure	interruptions intrinsic to surgical work	120	17.2
Environment	the ringing of the fixed-line telephone in the OR, noises of equipment alarms, messy operation tables, overly loud music, noise outside the OR	87	12.5
Electrophysiological monitoring staff	requesting to record number of electrodes	20	2.9
Nurses themselves	surgical count was too fast, CNs asked to check again, discrepancies between surgical count and instrument count paper, teaching SNs at the start of the learning curve	49	7.0
Overall		697	00

The first surgical counting interruptions affected CNs 336 times (48.2%), ORNs (including CNs and SNs) 243 times (34.9%) and SNs 118 times (16.9%). The overall distributions of the first surgical counting interruption sources were significantly different among CNs, SNs and ORNs (including CNs and SNs) (X^2^ = 154.515, *P* < 0.001) (Table 3).

**Table 3:** Frequency of circulating nurses, scrub nurses, operating room nurses(including circulating nurses and scrub nurses)affected by sources of the first surgical counting interruptions

Scource	CNs	SNs	ORNs	X^2^	P
People entering the OR (%)	64 (19.0%)	19 (16.1%)	10 (4.1%)	81.012	0.000
Surgeons (%)	21 (6.3%)	6 (5.1%)	5 (2.1%)	22.594	0.000
Anaesthetists (%)	26 (7.7%)	5 (4.2%)	4 (1.6%)	39.686	0.000
Instruments (%)	41 (12.2%)	24 (20.3%)	79 (32.5%)	49.563	0.000
Disinfection supply center (%)	39 (11.6%)	21 (17.8%)	57 (23.5%)	24.923	0.000
Procedure (%)	25 (7.4%)	30 (25.4%)	65 (26.7%)	31.200	0.000
Environment (%)	76 (22.6%)	6 (5.1%)	5 (2.1%)	171.414	0.000
Electrophysiological monitoring staff (%)	15 (4.5%)	2 (1.7%)	3 (1.2%)	23.550	0.000
Nurse themselves (%)	29 (8.6%)	5 (4.2%)	15 (6.2%)	26.694	0.000
Overall	336	118	243	154.515	0.000

CNs: circulating nurses, SNs: scrub nurses ORNs: including CNs and SN**s**

The results of multiple comparisons were significantly different. Compared with SNs and ORNs (including CNs and SNs), CNs were more affected by the first surgical counting interruptions (X^2^ = 77.618, *P* < 0.001; X^2^ = 12.775, *P* < 0.001) (Table 4).

**Table 4:** Frequency of circulating nurses, scrub nurses, operating room nurses(including circulating nurses and scrub nurses) affected by the first surgical counting interruptions

Roles	Influence	No influence	X^2^	P
CNs	336(48.2%)	361(41.8%)	77.618	0.000
SNs	118(16.9%)	579(83.1%)		
CNs	336(48.2%)	361(41.8%)	12.775	0.000
ORNs	243(34.9%)	454(63.1%)		
SNs	118(16.9%)	579(83.1%)	29.204	0.000
ORNs	243(34.9%)	454(63.1%)		

CNs: circulating nurses, SNs: scrub nurses ORNs: including CNs and SN**s**

In total, the study identified 697 interruption-associated 628 negative outcomes (90.1%) and 69 positive outcomes (9.9%) (Table 5). The overall distributions of interruption attributes included intrusions (*N* = 421, 60.4%), distractions (*N* = 127, 18.2%), discrepancies (*N* = 26, 3.7%), and breaks (*N* = 123, 17.7%) (Table 6). Intrusion was the major type of the first surgical counting interruption. We classified the responses to the first surgical counting interruptions into immediate interruptions (*N* = 446, 64.0%), slightly delayed interruptions (*N* = 113, 16.2%), refused interruptions (*N* = 33, 4.7%), and multitasking (*N* = 105, 15.1%) in this study (Table 7).

**Table 5:** Frequency of outcomes of the first surgical counting interruptions

outcomes	Number	Percentage (%)	
negative	628	90.1%	
positive	69	9.9%	

**Table 6:** Frequency of attributes of the first surgical counting interruptions

types	Number	Percentage (%)	
intrusion	421	60.4%	
distraction	127	18.2%	
discrepancy	26	3.7%	
break	123	17.7%	

**Table 7:** Frequency of responses to the first surgical counting interruptions

responses	Number	Percentage (%)	
immediate interruptions	446	64.0%	
slightly delayed	113	16.2%	
refused interruptions	33	4.7%	
multitasking	105	15.1%	

## Discussion

In this study, 697 surgical counting interruptions were recorded from 1015 surgical counts, with an average of 8.7 interruptions per hour. It has been previously reported that the frequency of interruptions was an average of 3–9.62 times per hour [10, 11]. However, this study revealed a greater level of interruptions. Surgical counting interruptions may prolong the process of surgical count. Because of time constraints, ORNs(including CNs and SNs) were rushed during surgical count. Human error may occur in manual count systems. It is dangerous for the patient and the surgical team. High-frequency interruptions may also increase stress, which may result in inferior technical performance [12, 13]. Therefore, these interruptions may affect the ability to identify hazards during surgery. Cognitive load theory views working memory as the primary bottleneck for learning, as it is limited in both retention and capacity [14]. Surgical count requires high working memory demands; therefore, interruption during surgical count may affect memory recall. Moreover, the first surgical examination is the initial phase of the procedure. Interruptions that accumulate over time reduce the compensatory resources of the ORNs (including the CNs and SNs), which may also affect the next procedure and safety of patients [15].

The observation data revealed that the main source of the first surgical counting interruptions was related to instruments (20.7%), followed by procedures (17.2%) and disinfection supply centres (16.8%). It should be noted that this finding may differ from other studies that suggested that the main sources of interruptions were entering/exiting the OR and communications [16, 17]. The difference may be related to different study phases and/or samples. Notably, we only investigated the first surgical count, while other studies have investigated the whole procedure. Interruptions induced by instruments have been rarely reported. We discovered that the factors related to checking the integrity of microsurgical instruments were the primary source of the first surgical counting interruptions. The development of microsurgical instruments parallels the growth of microsurgery, and microsurgical instruments will also be improved in accordance with doctors’ needs; both of these improvements will result in greater complexity in the design and use of such tools [18]. The microsurgical instruments that cause distress are the small assembly built-in screws and delicate tips. It is difficult and time-consuming to check the integrity of these materials. Surgical count may be performed under time pressure and safety pressure.

The study analysed the effects of the first surgical counting interruption on CNs, SNs and ORNs (including CNs and SNs) using observational data, and different nursing roles may be affected by differences in the sources of interruption. CNs were significantly more affected by the first surgical counting interruptions than SNs and ORNs (including CNs and SNs), which is consistent with prior research [15]. CNs and SNs are involved in surgical count, and the CN is usually an experienced nurse who plays an important role in surgical count. Some strategies, such as the implementation of safe zones, the Stay S.A.F.E. strategy are used to reduce interruptions [1, 19]. However, CNs cope with interruptions of the OR, surgical team and patient, and the nursing work environment is complex [20]. Therefore, there is no way to eliminate all interruptions in ORs. Surgical counting is often performed with a shortage of personnel, as it is a routine task that does not require increasing the number of team members [21]. Administrators should carefully consider optimizing staffing during chaotic stages and critical phases.

The results also showed that the majority of nurses who responded to the first surgical counting interruption immediately stopped their work. There may be three reasons for this. First, nurses regard interruption as an integrated part of clinical care. they are used to being interrupted at any time and in any situation. Therefore, they do not consider doing something to avoid it. Second, surgery is multidisciplinary. They had to take breaks immediately to coordinate with other team members. Third, the interruption may be positively related to the safety of the patient. Surgical count requires concentration, and interruptions may lead to distraction and influence the discovery of security threats [6, 22]. Managers should improve systems and processes to reduce unnecessary interruptions. Meanwhile, targeted strategies, such as training nurses to distinguish between detrimental and beneficial interruptions as well as perfecting their ability to respond to interruptions, may be effective [23, 24].We also found that multitasking was performed. Multitasking increases stress [25], thereby affecting the identification of surgical count hazards. Multitasking may be expressed as an integral part of daily practice and is inevitable, but it is important to create an environment where nurses can focus on critical phases to improve patient safety [26, 27].

## Conclusion

In conclusion, the frequency of the first surgical counting interruption is risky, and managers need to take steps to improve it. Although all counts were completed, no discrepancy was found at the end of the surgery. This may be within the range of resilient coping of ORNs (including CNs and SNs). The frequency of interruptions varies among surgical specialties and nursing roles (CNs and SNs), and the sources of interruptions also differ. Managers should consider the unique needs of each surgical specialty and nursing role (CNs vs. SNs) when developing interventions. Considering the supportive attributes of CNs and the complex working environment in ORs, interventions need to consider the support of systems and process improvements.

### Limitations

This study has several limitations. First, this study collected data through an observer’s review of surveillance video recordings, which may have resulted in some data loss due to human limitations such as attention span, distraction and memory of events. Second, our observations were limited to four disciplines of one hospital; therefore, the results may not be applicable to other hospitals. It is necessary for future research to enrol participants from hospitals at different levels and from more disciplines to improve sample representativeness. Third, this was an observational study. Future longitudinal studies or intervention studies that focus on the development of targeted strategies and the evaluation of their effectiveness are needed.

### Electronic supplementary material

Below is the link to the electronic supplementary material.

Supplementary Material 1

## Data Availability

The datasets generated and/or analysed during the current study are not publicly available due to privacy and ethical restrictions but are available from the corresponding author on reasonable request.
