# Supplementary material for: Surgical counting interruptions in operating rooms
Source: BMC Nurs. 2024 Apr 10;23:241. doi: 10.1186/s12912-024-01912-1 (PMC11008047; doi:10.1186/s12912-024-01912-1)
Supplement: Supplementary file 1 — Supplementary Material 1 [file 12912_2024_1912_MOESM1_ESM.docx]

**surgical counting interruption event form**

| Type  Role | Name | Gender | Age | Education | Years of working experience |
| --- | --- | --- | --- | --- | --- |
| SNs |  |  |  |  |  |
| CNs |  |  |  |  |  |

Note: SNs , scrub nurses, CNs ,circulating nurses

| Source | Type | Outcomes | Responses |
| --- | --- | --- | --- |
| ①People entering or exiting OR  ②Surgeons  ③Anesthetists  ④Instruments  ⑤Disinfection supply center  ⑥Procedure  ⑦Environment  ⑧Electrophysiological monitoring staff  ⑨Nurses themselves | ①Intrusion  ②Distraction  ③Discrepancy  ④Break | ①Negative type  ②Positive type | ①Immediate interruptions  ②Slightly delayed  ③Refused interruptions  ④Multitasking |
|  |  |  |  |

The first surgical count duration minutes

After all counts were complete and surgery was over:

Discrepancy: Yes No
